# Glucagon Like Peptide-1-Induced Glucose Metabolism in Differentiated Human Muscle Satellite Cells Is Attenuated by Hyperglycemia

**DOI:** 10.1371/journal.pone.0044284

**Published:** 2012-08-28

**Authors:** Charlotte J. Green, Tora I. Henriksen, Bente K. Pedersen, Thomas P. J. Solomon

**Affiliations:** The Centre of Inflammation and Metabolism at Department of Infectious Diseases and Copenhagen Muscle Research Centre, Rigshospitalet, The Faculty of Health Sciences, University of Copenhagen, Denmark; Mayo Clinic, United States of America

## Abstract

**Background:**

Glucagon like peptide-1 (GLP-1) stimulates insulin secretion from the pancreas but also has extra-pancreatic effects. GLP-1 may stimulate glucose uptake in cultured muscle cells but the mechanism is not clearly defined. Furthermore, while the pancreatic effects of GLP-1 are glucose-dependent, the glucose-dependency of its extra-pancreatic effects has not been examined.

**Methods:**

Skeletal muscle satellite cells isolated from young (22.5±0.97 yr), lean (BMI 22.5±0.6 kg/m^2^), healthy males were differentiated in media containing either 22.5 mM (high) or 5 mM (normal) glucose for 7 days in the absence or presence of insulin and/or various GLP-1 concentrations. Myocellular effects of GLP-1, insulin and glucose were assessed by western-blot, glucose uptake and glycogen synthesis.

**Results:**

We firstly show that the GLP-1 receptor protein is expressed in differentiated human muscle satellite cells (myocytes). Secondly, we show that in 5 mM glucose media, exposure of myocytes to GLP-1 results in a dose dependent increase in glucose uptake, GLUT4 amount and subsequently glycogen synthesis in a PI3K dependent manner, independent of the insulin signaling cascade. Importantly, we provide evidence that differentiation of human satellite cells in hyperglycemic (22.5 mM glucose) conditions increases GLUT1 expression, and renders the cells insulin resistant and interestingly GLP-1 resistant in terms of glucose uptake and glycogen synthesis. Hyperglycemic conditions did not affect the ability of insulin to phosphorylate downstream targets, PKB or GSK3. Interestingly we show that at 5 mM glucose, GLP-1 increases GLUT4 protein levels and that this effect is abolished by hyperglycemia.

**Conclusions:**

GLP-1 increases glucose uptake and glycogen synthesis into fully-differentiated human satellite cells in a PI3-K dependent mechanism potentially through increased GLUT4 protein levels. The latter occurs independently of the insulin signaling pathway. Attenuation of both GLP-1 and insulin-induced glucose metabolism by hyperglycemia is likely to occur downstream of PI3K.

## Introduction

Glucagon like peptide-1 (GLP-1) is a potent incretin hormone produced by L-cells in the ileum and colon. GLP-1's most well defined function is in the pancreas where it augments glucose-stimulated insulin secretion [1]. Extra-pancreatic effects of GLP-1 have also been described. Peripherally, GLP-1 is known to affect gut motility [2], [3], and centrally GLP-1 induces satiety [4], [5]. Thus, GLP-1 is of great clinical interest for the treatment of metabolic diseases.

Recent work has suggested that GLP-1 may also have an extra-pancreatic effect in skeletal muscle. Recently it has been shown in rats that GLP-1 increases glucose use and microvasculature in muscle [6] and that this is attenuated by the GLP-1 antagonist exendin-9. The multiple actions of GLP-1 are thought to be mediated through a single G-protein coupled receptor, the GLP-1 receptor (GLP-1R). Binding studies have been carried out on the GLP-1R [7], [8] in rat muscle models; however, to our knowledge, no investigation into the receptor protein expression in human skeletal muscle has been carried out. Furthermore, the evidence that GLP-1 plays a role in skeletal muscle metabolism emanates almost exclusively from the laboratory of Villanueva-Penacarillo. This group has reported that GLP-1 promotes glucose transport and glycogen synthase activity in rat and human skeletal muscle via a protein kinase C (PKC) related mechanism [9]–[12]. Indeed, Acitores et al [10] report that in rat muscle low doses of GLP-1 increased phosphoinositide 3-kinase (PI3K) activity and phosphorylation of protein kinase B (PKB/Akt), 70 kDa ribosomal protein S6 kinase (p70S6K) and extracellular signal-regulated kinase (ERK). However, the mechanism by which GLP-1 promotes glucose metabolism in muscle is poorly defined due to the use of a number of unspecific inhibitors. In particular the PKC inhibitor, RO318220, was shown to significantly inhibit GLP-1-induced glucose metabolism. However, in 1996 Alessi et al [13] showed that RO318220 also potently inhibits p90 ribosomal protein S6 kinase 2 (RSK2) and p70S6K (and at higher concentrations PKB/Akt). Therefore, it is difficult to interpret the conclusions of Acitores et al regarding PKCs role in GLP-1 mediated muscle glucose transport.

In a subsequent study from the same group [12], GLP-1 mediated glucose transport in human myocytes was confirmed. This work also defines the role of PKC by using high dose RO318220, however, conversely to their prior work, no effect on GLP-1 (or insulin) induced glucose uptake was found. A second PKC inhibitor, H-7, was also used. H-7 is a potent protein kinase inhibitor (Ic50 PKA = 3 µm, PKG  = 5.7 µM and PKC = 6 µM) [14] which suppressed GLP-1 mediated glucose transport [10]. While not discussed by the authors, this suggests a protein kinase A (PKA) or protein kinase G (PKG)-dependent mechanism. The lack of consistency of the methodologies and findings, and the varying concentrations of GLP-1 (from 0.1–10 nM) used in these prior studies makes the interpretation of the results difficult. Therefore more structured and focused research needs to be carried out in skeletal muscle examining both the functional role of GLP-1 and the expression of the GLP-1R.

**Figure 1:**
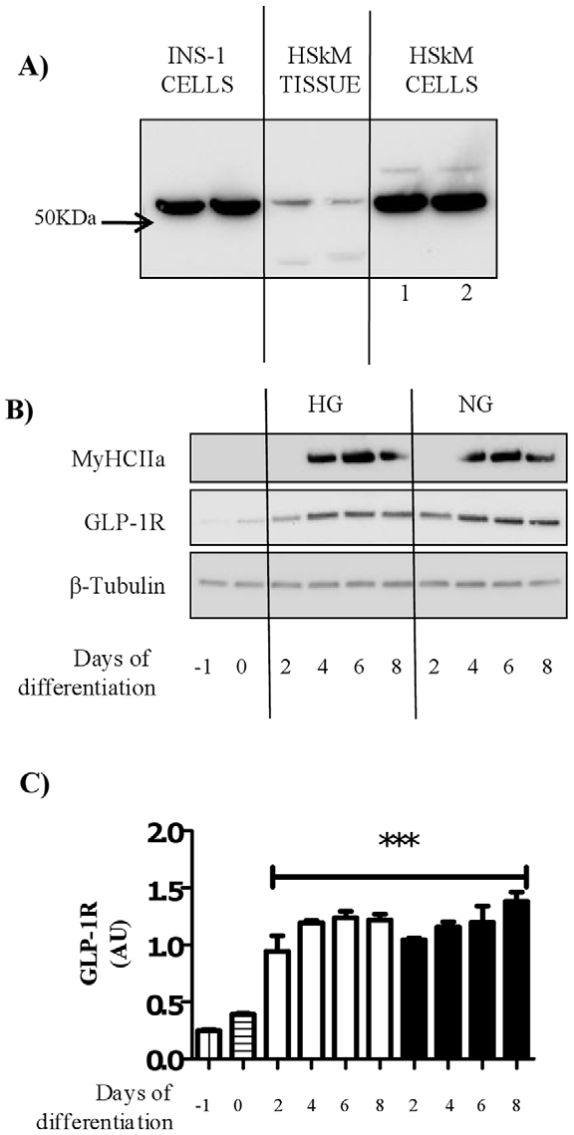
GLP-1 receptor protein expression in human skeletal muscle tissue and human myocytes. (A) Protein lysate (20 µg) from either INS-1 cells, human skeletal muscle tissue or human myocytes (fully differentiated satellite cells) were immunoblotted to assess the protein expression of the GLP-1 receptor (GLP-1R). A band was visualized at approximately 53 kDa in INS-1 cells corresponding to the GLP-1R. This band was also detected in both human muscle tissue and myocytes. The GLP-1R expression in myocytes was assessed when cells were differentiated in 22.5 mM (lane 1) and 5 mM (lane 2) glucose. (B) Satellite cell protein was collected when cells fully confluent (Day −1), after alignment (day 0) and after 2, 4 6 and 8 days of differentiation in either high (22.5 mM) or normal glucose (5 mM) media. Total protein lysate was immunoblotted to assess the expression of GLP-1 receptor and Myosin heavy chain 2a (MyHC2a). Equal protein was ascertained by immunoblotting with an antibody against β-tubulin. (C) Quantification of GLP-1 receptor during and differentiation (n = 4), bars represent fully confluent cells (day −1), aligned cells (day 0) and cells after 2, 4, 6 and 8 days of differentiation in either high (open bars) or normal glucose (black bars) media. GLP-1 receptor expression expressed normalized to total protein and expressed as arbitrary units. Significant changes from day −1 and day 0 are indicated by *** (P<0.0005).

It is well-described that the insulin-secretory pancreatic effect of GLP-1 occurs when glucose is transiently raised above basal levels (e.g. postprandial state). However, in hyperglycemic individuals (e.g. type 2 diabetics), GLP-1 stimulation of insulin is poor [15]. Similarly, in hyperglycemic individuals skeletal muscle insulin sensitivity of glucose uptake is also poor [16]. Thus, because hyperglycemia has been shown to alter both insulin responsiveness in skeletal muscle and GLP-1 responsiveness in the pancreas it is essential that GLP-1 mediated glucose uptake in a cell culture model is characterized in both normal and high glucose levels. While previous work investigating GLP-1 effects on muscle have examined diabetic muscle from rats [17], [18] and myocytes from diabetic humans [12], the causality of hyperglycemia has not been examined.

**Figure 2:**
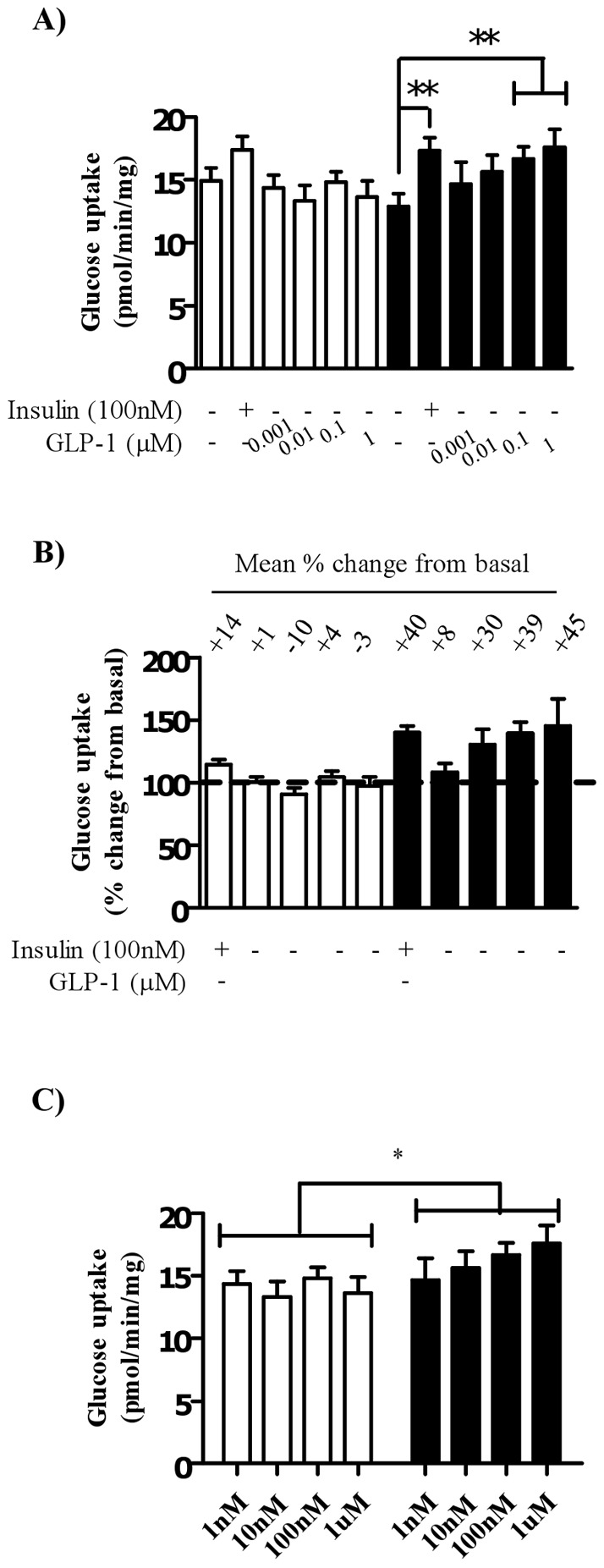
Effect of GLP-1 treatment on glucose uptake into myocytes cultured in high or normal glucose media. Satellite cells were isolated from lean, healthy males (n = 6), differentiated in either high glucose (22 mM, open bars) containing media or normal glucose (5 mM, black bars) containing media for seven days. A) Myocytes were treated with either 100 nM insulin or GLP-1 at indicated concentrations for 30 minutes before assessing 2-deoxyglucose uptake. B) Glucose uptake was expressed as a percent change from basal levels for either high (open bars) or normal (black bars) glucose conditions. C) Myocytes treated with GLP-1 concentrations only in both glucose conditions were subjected to a two-way ANOVA. ** denotes a significant change (P<0.005) from basal levels. * denotes a significant difference (P<0.05) between glucose conditions.

If GLP-1-mediated glucose uptake in healthy skeletal muscle occurs via a mechanism similar to that of insulin, it can also be hypothesized that hyperglycemia may also result in impaired GLP-1-induced glucose uptake in human skeletal muscle. In an attempt to understand the role of GLP-1 in human skeletal muscle and subsequently if this is affected by hyperglycemia, human muscle satellite cells isolated from healthy individuals were differentiated in either high (22.5 mM) or normal (5 mM) glucose conditions and the effects of GLP-1 (and insulin) on glucose metabolism and insulin signaling were assessed.

**Figure 3:**
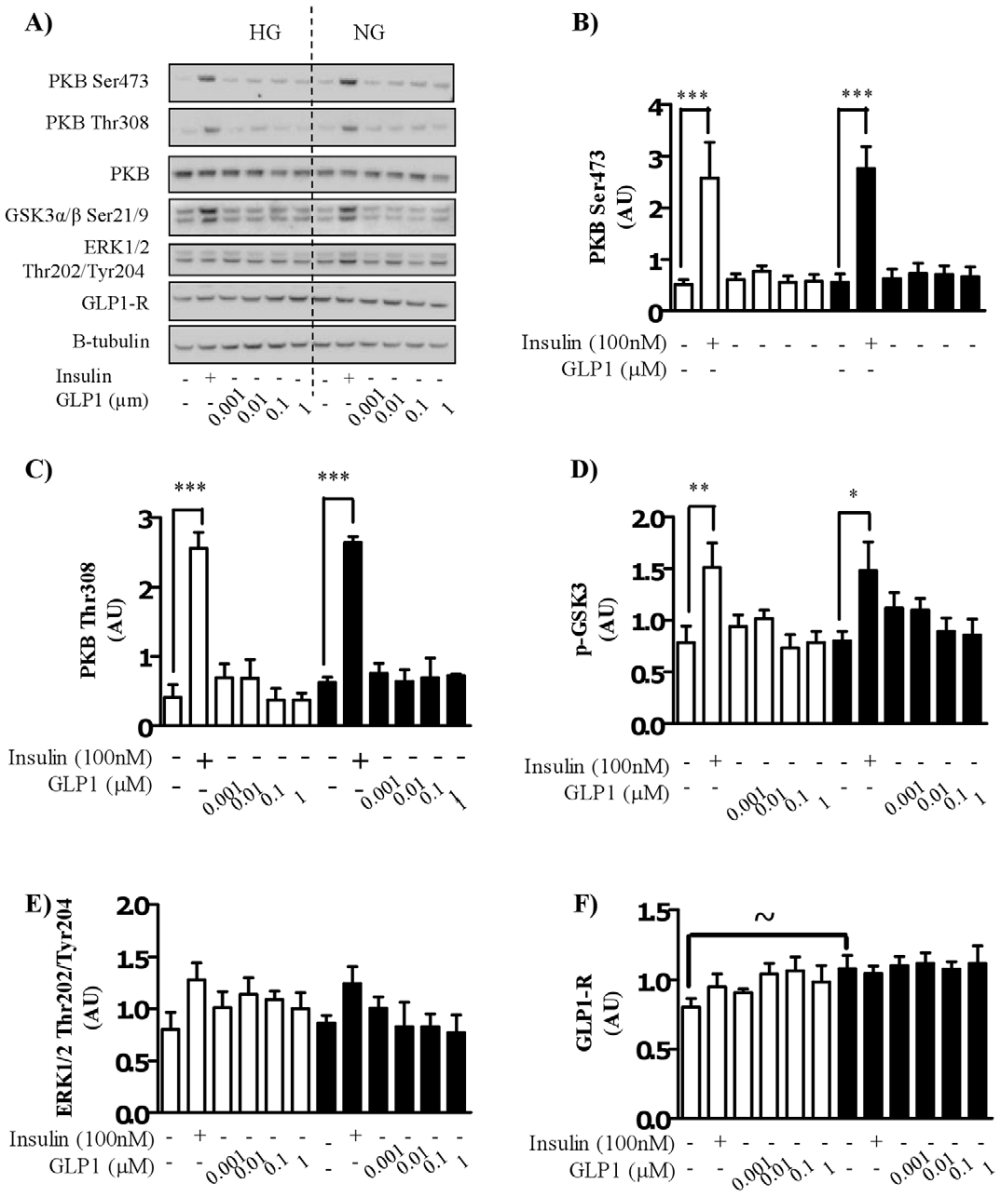
Effect of GLP-1 treatment on the insulin signaling cascade in myocytes cultured in high or normal glucose media. Satellite cells were isolated from lean, healthy males (n = 6), and differentiated in either high glucose (22 mM) or normal glucose (5 mM) media for seven days. Myocytes were treated with either 100 nM insulin or GLP-1 at indicated concentrations for 30 minutes before A) immunoblotting to assess the phosphorylation status of PKB/Akt, GSK3 and ERK1/2 and the total protein abundance of PKB/Akt and GLP-1R. Equal loading was ascertained by immunoblotting with an antibody against β-tubulin. Effect of high (open bars) and normal (black bars) glucose on phosphorylation of PKB Ser473 (B), PKB Thr308 (C), GSK3 (D) and ERK (E), and the total protein amount of GLP-1R (F) induced by insulin and GLP-1 was quantified (n = 6), normalized to total protein and expressed as arbitrary units. Significant changes from basal levels are indicated by * (P<0.05), ** (P<0.001), and *** (P<0.0001). Significant differences between normal and high glucose in the basal state is indicted by ∼ (P<0.05).

## Experiments

### Subjects

This study was performed in accordance with the Helsinki II Declaration and was approved by the Scientific Ethics Committee of the Capital Region of Denmark. Skeletal muscle biopsies (N = 6) from the *vastus lateralis* were obtained by the percutaneous needle biopsy method under local anesthesia from young (22.5±0.97 yr), lean (BMI 22.5±0.6 kg/m^2^), healthy men. All participants provided informed, written consent before participation.

**Figure 4:**
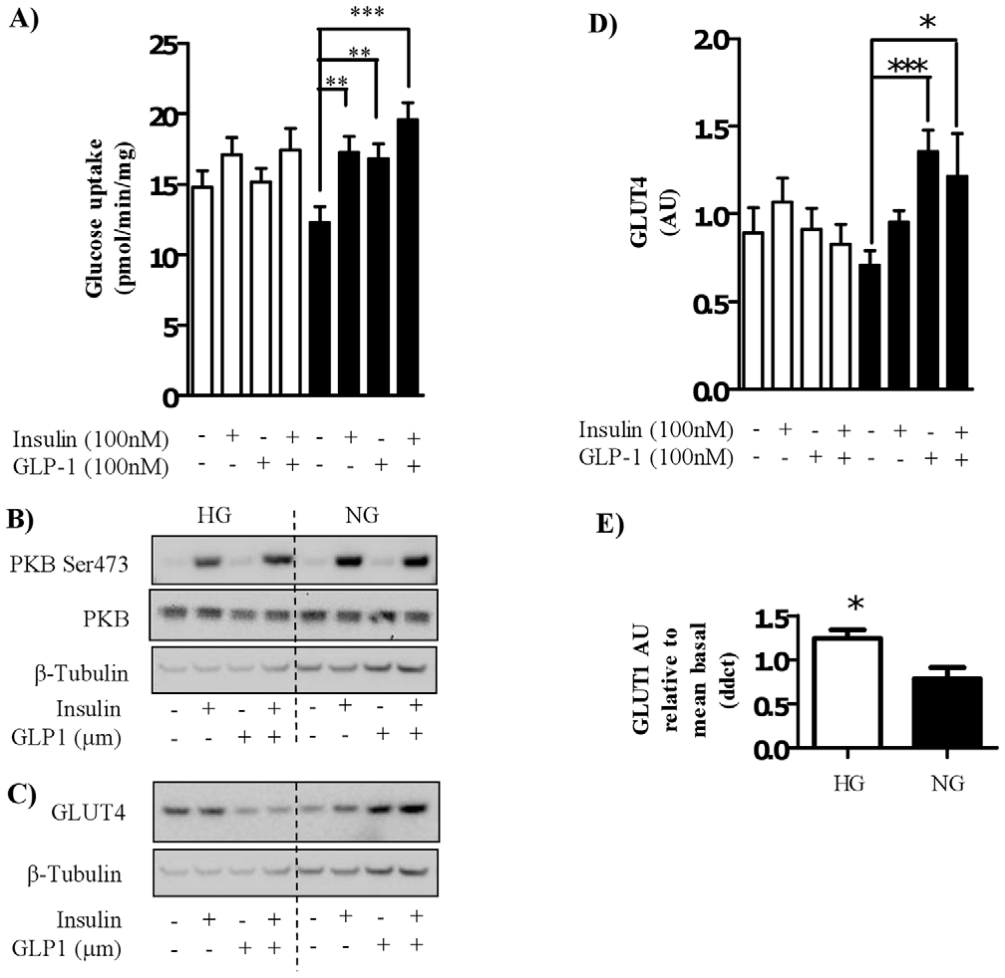
Effect of GLP-1 and/or insulin on glucose uptake and GLUT4 protein. Satellite cells were isolated from lean, healthy males (n = 6), and differentiated in either high glucose (22 mM, open bars) or normal glucose (5 mM, black bars) media for seven days. Myocytes were treated with either 100 nM insulin and/or 100 nM GLP-1 for 30 minutes before either (A) assessing 2-deoxyglucose uptake or (B) immunoblotting to assess phosphorylation status of PKB/Akt and total protein amount of PKB/Akt or (C) immunoblotting to assess the protein level of GLUT4. The effect of high (open bars) and normal (black bars) glucose on GLUT4 protein level was quantified ((D); n = 6), normalized to total protein and expressed as arbitrary units. Significant changes from basal levels are indicated by * (P<0.05), ** (P<0.001), and *** (P<0.0001). (E) The effect of high glucose on GLUT1 mRNA expression in the basal state was measured by qPCR (n = 4). The significant effect of high (open bars) glucose on GLUT1 expression relative to normal (black bars) glucose is indicated by * (P<0.05).

### Materials

F10 nutrient mixture (HAM), DMEM (Dulbecco's Modified Eagle's Medium) low and high glucose, FBS (Fetal Bovine Serum), HS (Horse Serum), fungizone antimycotic (FZ), and penicillin/streptomycin (P/S) were all from Invitrogen (Taastrup, DK). Insulin (Actrapid) was from Novo Nordisk (Bagsværd, DK). GLP-1 was from poly-peptide group (Hillerød, DK). Wortmannin was from MERCK (Darmstadt, DE). Complete (mini) protein phosphatase inhibitor tablets were purchased from Boehringer-Roche Diagnostics (Copenhagen, DK) and protein protease inhibitor I and II were from Sigma-Aldrich (Brøndby, DK). 2-deoxy-D-[^3^H]-glucose and [^14^C]-U-glucose were from Perkin Elmer Life Sciences (Copenhagen, DK). Phospho-Akt/PKB (Ser^473^), phospho-Akt/PKB (Thr^308^), phospho-GSK3α/β (Ser^21/9^), phospho-ERK1/2 (Thr^202^/Tyr^204^), phospho-PKCζ/λ (Thr^410/403^), anti-PKB and anti-β-tubulin, antibodies were from Cell Signalling Technology (Boston, MA). Anti-GLP-1-receptor was from Abcam (Cambridge, UK); anti-GLUT4 was from Thermo scientific (Soeborg, DK); and anti-myosin heavy chain 2a (MF20) was from Developmental Studies Hybridoma Bank (University of Iowa). TRIzol was from Invitrogen (Taastrup, DK).

**Figure 5:**
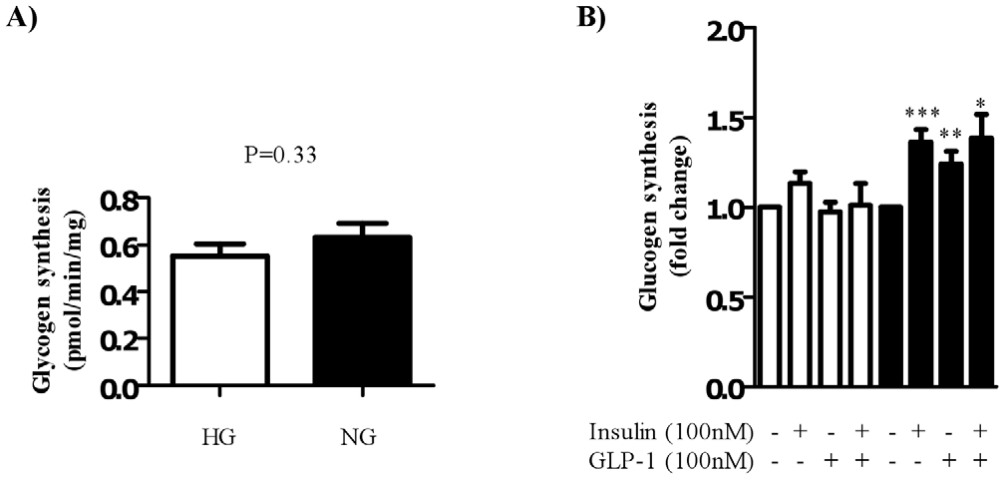
Effect of GLP-1 treatment on glycogen synthesis in myocytes cultured in high or normal glucose media. Satellite cells were isolated from lean, healthy males (n = 6), and differentiated in either high glucose (22 mM, open bars) or normal glucose (5 mM, black bars) media for seven days. Myocytes were treated with either 100 nM insulin and/or 100 nM GLP-1 for 30 minutes before assessing glycogen synthesis (by measuring incorporation of [^14^C]-glucose in to glycogen). (A) Shows absolute rates of glycogen synthesis in the basal level (n = 6, in triplicate); (B) indicates fold changes from basal levels for each glucose condition. Significant changes from basal levels are indicated by * (P<0.05), and ** (P<0.001).

### Human muscle satellite cell proliferation and differentiation

Muscle satellite cells were isolated from *vastus lateralis* muscle biopsies as previously described [19]. To minimize fibroblast contamination, cells were pre-seeded in a culture dish for 3 hours in F10/HAM, 20% Fetal bovine serum (FBS), 1% penicillin/streptomycin (PS), 1% Fungizone. Unattached cells were then removed and seeded into a culture flask, pre-coated with matrigel (BD Biosciences, San Jose, CA, USA). Following 4 days of incubation, the cell culture medium was changed and then every second day thereafter. 100% confluent cells (day −1 in figures) were incubated with DMEM containing 1 g/l glucose, 10% FBS, 1% penicillin/streptomycin (P/S) in order to allow cells to align, after 2 days of alignment (day 0 in figures) differentiation was initiated by changing the media to DMEM containing either 4.5 g/l (22.5 mM) or 1 g/l (5 mM) glucose supplemented with 2% horse serum (HS), 1% P/S. For all experiments, cells were used at day 6 or 7 of differentiation (and at this point are termed myocytes) at either passage 4 or 5. All six cell IDs were used on the same day of differentiation and at the same passage number for each experimental data set and each cell ID underwent experiments in both glucose conditions.

**Figure 6:**
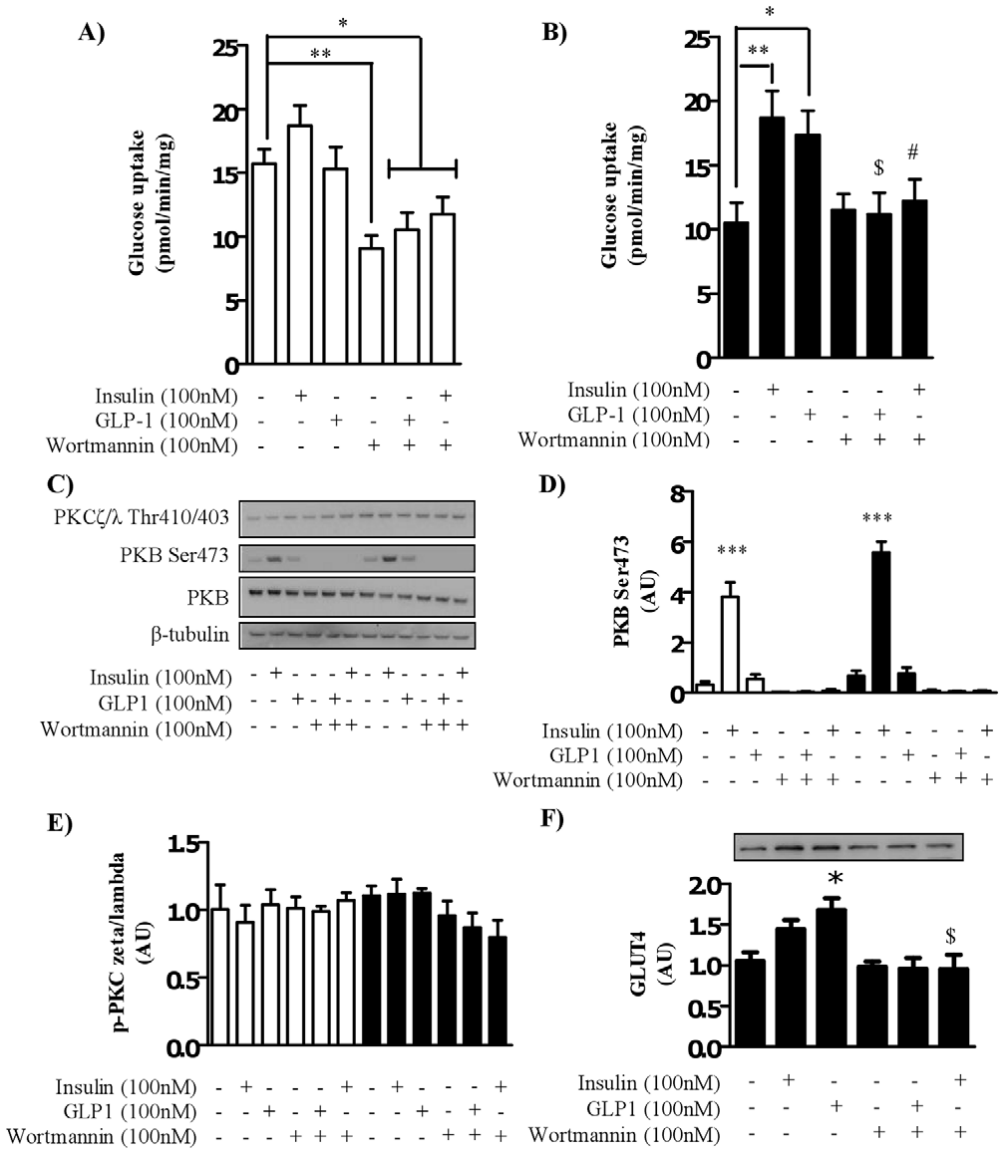
The effect of GLP-1 on glucose uptake in myocytes cultured in normal glucose media is PI3K-dependent. Satellite cells were isolated from lean, healthy males (n = 6), and differentiated in either high glucose (22 mM, open bars) or normal glucose (5 mM, black bars) media for seven days. Myocytes were treated with 100 nM wortmannin (40 minutes) in the absence or presence of either 100 nM insulin or 100 nM GLP-1 for the penultimate 30 minutes before assessing 2-deoxyglucose uptake in to myocytes (A) cultured in high glucose (22 mM) or (B) normal glucose (5 mM) media. (C) Lysates were immunoblotted for phosphorylation status of PKB/Akt and PKCζ/λ and total protein amount of PKB/Akt under the same conditions. Immunoblots were quantified (n = 6), normalized to total protein and expressed as arbitrary units (D and E). (F) Total protein amount of GLUT4 was assessed by immunoblot and quantified (n = 5). Significant changes from basal levels are indicated by * (P<0.05), ** (P<0.001), and *** (P<0.0001). A # denotes a significant change (P<0.05) from insulin treatment and a $ denotes a significant change (P<0.05) from GLP-1 treatment.

### Cell Lysis

Cells were rinsed once in ice-cold phosphate buffered saline (PBS) and lysed in (20 mM Tris, pH 7.5, 150 mM NaCl, 1 mM EGTA, 1 mM EDTA, 0.1% Triton X-100, protease inhibitor (1 tablet/10 ml), 1% phosphatase inhibitor cocktail). Whole cell lysates were centrifuged (12000 *g* at 4°C for 10 min), and supernatants removed for storage at −80°C until required.

### Immunoblotting

Protein concentrations were determined using the Bradford reagent [20]. 20 μg whole cell lysates were subjected to SDS-PAGE using Invitrogen 4-12% precast gels and I-blot dry transfer machine according to manufacturer's instructions. PVDF membranes were probed with primary antibodies raised against the protein of interest as indicated in the figure legends. Detection of primary antibodies was performed using appropriate peroxidase-conjugated IgG and protein signals visualized using FEMTO enhanced chemiluminescence and Biorad Chemidoc XRS imager. Quantification of immunoblots was done using Image J software (NIH, Bethesda, MD, http://rsb.info.nih.gov/ij).

**Figure 7:**
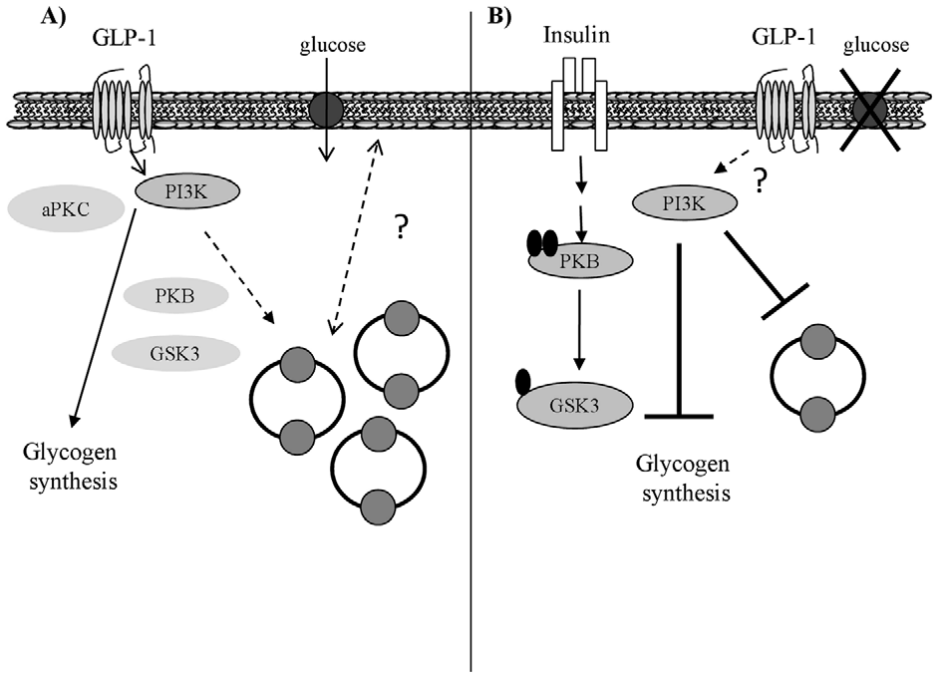
Schematic diagram illustrating the effects of GLP-1 and insulin on skeletal muscle glucose metabolism under normal glucose conditions and during hyperglycemia. A) GLP-1 effects: Under normal glucose conditions, we propose that GLP-1 signals through its receptor promoting PI3K activation and an increased level of GLUT4 protein (potentially through an increased synthesis of GLUT4 storage vesicles) in the cytoplasm resulting in increased glucose uptake into the cell. GLP-1 stimulates glucose uptake independently of PKB phosphorylation and promotes glucose storage independently of GSK3 phosphorylation. GLP-1 also has no effect on phosphorylation of atypical PKC's. B) Hyperglycemia effects: Hyperglycemia had no effect on insulin-stimulated PKB or GSK3 phosphorylation but attenuates insulin-mediated glucose uptake and subsequently glycogen synthesis. Hyperglycemia is unlikely to suppress GLP-1-induced PI3K activity; however it significantly suppresses GLP-1 induced increases in GLUT4 protein levels. Following from this hyperglycemia blocks GLP-1-induced glucose uptake into the cells and resultant glycogen synthesis.

### RNA isolation and quantitative real time PCR

Total RNA was isolated from cells using TRIzol according to manufacturer's instructions. Reverse transcription and cDNA synthesis was performed as previously described [21]. Primers for GLUT1 were designed using NCBI blast and synthesized by DNA technology (Risskov, DK). GLUT1 forward primer: 5′ - ACCGGGCCAAGAGTGTGCTA-3′, reverse primer: 5′ -GTAGGCGGGGGAGCGGAACA-3′. 18S was amplified using pre-developed assay reagents (Applied Biosystems) and used as a reference gene. The primers were mixed with cDNA and SYBR®GREEN PCR Master Mix (Applied Biosystems) in a total reaction volume of 10 μl. Detection of mRNA expression was performed in triplicate using a Viia7 sequence detector (Applied Biosystems). To adjust for variations in the cDNA synthesis each gene was normalized to that of 18 S ribosomal RNA using the comparative (ΔΔCT) method.

### Glucose uptake

Myocytes were serum starved in DMEM containing either 4.5 g/l or 1 g/l glucose for 2 hours prior to assaying glucose uptake and incubated with reagents for times and at concentrations indicated in the figure legends. Cells were washed twice with Hepes-buffered saline (140 mM NaCl, 20 mM Hepes, 5 mM KCl, 2.5 mM MgSO_4_ and 1 mM CaCl_2_, pH7 4). Glucose uptake was assayed by incubation with 10 μM 2-deoxy-D-[^3^H]glucose (1 μCi/ml) for 10 min. Non-specific tracer binding was determined by quantifying cell-associated radioactivity in the presence of 10 μM cytochalasin B. Medium was aspirated before washing adherent cells twice with 0.9% ice cold NaCl. Cells were subsequently lysed in 50 mM NaOH and radioactivity was quantified using a Perkin Elmer Tri-Carb 2810TR scintillation counter. Protein concentration was determined using the Bradford reagent.

### Glycogen synthesis

Myocytes were serum starved in DMEM containing either 4.5 g/l or 1 g/l glucose for 2 hours prior to assaying glycogen synthesis. Cells were washed twice with Hepes-buffered saline (140 mM NaCl, 20 mM Hepes, 5 mM KCl, 2.5 mM MgSO_4_ and 1 mM CaCl_2_, pH7 4) before addition of glucose oxidation buffer (5 mM glucose, 2 µCi/ml [^14^C]-U-glucose, in HBS pH 7.4). Cells were incubated at 37^o^C, 5% CO_2_ for 2 hours. Cells were washed twice in HBS buffer (pH7 4) and lysed in 50 mM NaOH. Lysates were boiled for 30 min before addition of 5 mg/ml glycogen and 95% ethanol, and glycogen was precipitated overnight (4°C). The precipitate was centrifuged at 13000 g at 4°C for 30 mins and glycogen was re-precipitated for 2 hours at −20°C. The glycogen pellet was resuspended in H_2_O and radioactivity was quantified using a Perkin Elmer Tri-Carb 2810TR scintillation counter. Protein concentration was determined using the Bradford reagent.

### Statistical analyses

For multiple comparisons statistical analysis was performed using one-way or two-way analysis of variance (ANOVA) with Bonferroni corrections. For data that was normalized to basal (i.e. fold or percent changes) statistical analysis was performed using a one-sample t-test. Data analysis was performed using GraphPad Prism software (GraphPad, San Diego, CA) and considered statistically significant at P values <0.05.

## Results

### GLP-1 receptor protein is expressed in human skeletal muscle tissue and differentiated muscle satellite cells

In order to assess whether human skeletal muscle expresses the GLP-1 receptor (GLP-1R), INS-1 cells were used as a control in the immunoblot. INS-1 cells are a pancreatic beta-cell line known to express a functional GLP-1R (predicted molecular weight 53 kDa). A band corresponding to 53 kDa was detected in both human skeletal muscle tissue and fully differentiated satellite cells (hereon referred to as myocytes). This band corresponded to that observed in the INS-1 cell lysate (Figure 1A). It should be noted that the relative expression of GLP-1R in myocytes was comparable to that seen in INS-1 cells. Furthermore, GLP-1R expression was not different between the high and normal glucose culture conditions. In order to fully characterize the expression of the GLP-1 receptor in myocytes, lysate from cells at various points in their differentiation were subjected to immunoblot. GLP-1 receptor protein was present in all samples however, its expression was significantly increased in differentiating cells (Figure 1 B and C) compared to fully confluent (day −1) or aligned cells (day 0). This increase in GLP-1R expression was not affected by glucose content of the differentiation media (Figure 1C) and appears to precede the expression of myosin heavy chain 2a (MyHCIIa) protein. Differentiation of cells in high or normal glucose did not significantly affect protein expression of MyHCIIa (P = 0.46 on day 6 of differentiation, n = 4).

### GLP-1 dose dependently increases glucose uptake into human myocytes, an effect that is attenuated under hyperglycemic media conditions

Myocytes differentiated under normal glucose (5 mM) conditions exhibited characteristic insulin-stimulated glucose uptake (approximately 40% above basal), this effect was almost completely lost in cells differentiated in high glucose (22.5 mM) conditions: approximately 14% increase from basal (Figure 2A and B). It should also be noted that in myocytes differentiated in high glucose (hyperglycemic) conditions there was a trend towards increased basal glucose uptake compared with cells differentiated in normal glucose conditions. A dose of either 100 nM or 1 µM GLP-1 significantly increased glucose uptake (approximately 39% and 45% respectively) in myocytes differentiated in normal glucose containing media. Importantly, differentiation of satellite cells in high glucose media also significantly inhibited GLP-1-induced glucose uptake at all doses (Figure 2A, B). When GLP-1 treatment in both glucose conditions was analyzed using a two-way ANOVA there was a significant effect of glucose on GLP-1 treatment (P = 0.02, Figure 2C).

### PKB/Akt and GSK3 phosphorylation are unaffected by hyperglycemic conditions or by GLP-1

Despite attenuation of insulin-stimulated glucose uptake under hyperglycemic conditions insulin significantly induced phosphorylation of PKB/Akt (Figure 3A) at Ser^473^ (Figure 3B) and Thr^308^ (Figure 3C) and that of downstream target, glycogen synthase kinase 3 (GSK3α/β) (Figure 3A and D) under both glucose conditions. There was no significant difference between high glucose and normal glucose in terms of phosphorylation of these proteins by insulin. Interestingly, GLP-1, unlike insulin had no effect on the phosphorylation of PKB/Akt or GSK3α/β at any dose in either glucose condition. In both glucose conditions, neither insulin, nor GLP-1, significantly increased the phosphorylation of the MAP-kinase, ERK1/2 (Figure 3A and E). Any changes in PKB/Akt phosphorylation occurred independently of changes in total PKB/Akt levels (Figure 3A) and there was no significant difference between groups. Total protein expression of the GLP-1R was significantly decreased in the basal state under high glucose conditions compared to normal glucose but was not significantly different in any other condition and was unaltered by either insulin or GLP-1 treatment (Figure 3A and F).

### GLP-1 does not significantly enhance insulin-stimulated glucose uptake

In myocytes differentiated in normal glucose conditions, both insulin and GLP-1 significantly increased glucose uptake. In myocytes treated with a combination of insulin and GLP-1 there was a trend to enhanced glucose uptake above levels observed with insulin or GLP-1 alone (approximately 15% higher than observed with insulin or GLP-1 alone) however, the difference between these treatments did not reach significance (Figure 4A). As described earlier, myocytes differentiated in high glucose conditions showed no GLP-1-induced glucose uptake and a robust suppression in insulin-stimulated glucose uptake. Additionally, in line with earlier findings (Figure 3A and B), PKB/Akt Ser^473^ phosphorylation was increased in both high and normal glucose conditions by insulin and there was no further increase observed in myocytes treated with insulin and GLP-1 in combination (Figure 4B). Changes in PKB/Akt phosphorylation were independent of changes in total PKB/Akt levels.

### GLP-1 increases the protein level of GLUT4 in human myocytes

In order to further understand the effects of GLP-1 on glucose metabolism in myocytes, the level of GLUT4 protein was measured in total protein lysates. In myocytes differentiated in normal glucose media, GLP-1 caused a significant increase in total GLUT4 protein levels (Figure 4C) resulting in an approximate 50% increase in GLUT4 protein compared to the amount quantified in basal cells (Figure 4D). This effect of GLP-1 was also attenuated in myocytes differentiated under hyperglycemic conditions. Under both glucose conditions, insulin did not significantly increase GLUT4 protein levels and combination treatment of GLP-1 and insulin did not further enhance GLUT4 levels above that observed with GLP-1 treatment alone. Differentiation of cells in high glucose had no significant effect on GLUT4 protein level compared to cells differentiated in normal glucose (P = 0.3). Interestingly, differentiation of cells in high glucose media significantly increased mRNA expression of GLUT1 compared to basal levels (Fig. 4E).

### Differentiation of myocytes under hyperglycemic conditions inhibits both insulin and GLP-1-mediated glycogen synthesis

In order to assess the functional consequence of GLP-1-induced glucose uptake, glycogen synthesis was measured in myocytes differentiated in high and normal glucose conditions. There was no significant effect on basal glycogen synthesis rate in cells differentiated under hyperglycemic conditions when compared to normal glucose. As there was variation between samples, glycogen synthesis was expressed as a fold change from basal for each cell ID used. In myocytes differentiated in normal glucose containing media, both insulin and GLP-1 robustly increased glycogen synthesis at the same dose (100 nM) that promoted enhanced glucose uptake (Figure 5B) approximately 1.5 and 1.25 fold change respectively. Combination treatment of insulin and GLP-1 did not significantly enhance the glycogen synthesis rate observed with the individual treatments alone. Importantly, in line with our finding that high glucose conditions inhibit both insulin and GLP-1-mediated glucose uptake, myocytes differentiated in high glucose media had a significantly suppressed glycogen synthesis response to both insulin and GLP-1.

### GLP-1-induced glucose uptake is PI3K dependent and PKB/Akt independent

To understand the mechanism by which GLP-1 mediates glucose uptake in myocytes we pharmacologically inhibited a critical component of the insulin signaling cascade, PI3K. Elevated basal glucose uptake levels in myocytes differentiated under high glucose conditions (P = 0.01, Fig 6 A and B) were significantly suppressed by treatment of myocytes with the PI3K inhibitor, wortmannin (Figure 6A). Consistent with this, all myocytes differentiated in hyperglycemic conditions that were treated with wortmannin exhibited significantly suppressed glucose uptake levels than that measured in basal samples. Importantly, wortmannin significantly attenuated both GLP-1 and insulin-induced increases in glucose uptake in myocytes differentiated under normal glucose conditions (Figure 6B). In normal glucose conditions, wortmannin treatment alone (or in the presence of insulin or GLP-1) did not significantly suppress glucose uptake compared to basal levels. Consistent with earlier findings, insulin significantly increased PKB/Akt Ser^473^ phosphorylation under both glucose conditions. The changes in PKB/Akt Ser^473^ phosphorylation occurred independently of changes in total PKB/Akt levels (no significant difference was found between PKB levels in myocytes differentiated in high or normal glucose media, data not shown). Additionally, GLP-1 did not have a significant effect on the level of PKB/Akt phosphorylation when compared to basal in either glucose conditions (Figure 6C and D). Wortmannin suppressed basal PKB/Akt phosphorylation and significantly attenuated insulin-induced PKB/Akt phosphorylation in both experimental glucose conditions. In an attempt to define the mechanism of GLP-1-mediated glucose uptake downstream of PI3K, we measured the phosphorylation of atypical PKCζ/λ. GLP-1 had no effect on PKCζ/λ phosphorylation in either glucose condition (Figure 6C and E) and there was no significant effect of hyperglycemia on basal PKC phosphorylation and inhibition on PI3K using wortmannin did not suppress PKC phosphorylation below basal levels in either glucose condition. Insulin had no effect on PKC phosphorylation in cells differentiated in either normal or high glucose media. These findings suggest that atypical PKCs are an unlikely candidate for the mechanism by which GLP-1 promotes glucose metabolism in skeletal muscle. In order to support a GLP-1-PI3K-GLUT4 dependent mechanism GLUT4 protein level was measured in cells treated with GLP-1 and/or wortmannin. In normal glucose conditions, wortmannin significantly attenuated GLP-1-mediated increases in GLUT4 protein level (Fig 6F).

## Discussion

Our findings indicate that under normal glucose (5 mM) conditions, GLP-1 promotes glucose uptake into myocytes, increases glycogen synthesis (Figure 5) and increases protein level of GLUT4 in total cell lysate (Figure 4C and D). Importantly, we have shown that GLP-1-mediated glucose transport is PI3K dependent (Figure 6B) and independent of key mediators of the insulin signaling pathway. Our results support previous findings that differentiating muscle satellite cells in high glucose media inhibits insulin-stimulated glucose transport and glycogen synthesis [22], [23]. Additionally, we provide novel data showing that GLP-1-mediated glucose transport and glycogen synthesis in myocytes are inhibited under high glucose (22.5 mM) conditions. The actions of GLP-1 are thought to be mediated through a single G-protein coupled receptor, the GLP-1 receptor (GLP-1R). Prior investigations into GLP-1 effects in skeletal muscle have not studied the GLP-1R, and are therefore incomplete. To our knowledge this is the first study to show that GLP-1R is expressed, at the protein level, in human myocytes and skeletal muscle tissue. Although expression of the receptor is relatively less in skeletal muscle tissue compared to cells its presence would suggest a physiological relevance. We hypothesize that GLP-1R is predominately expressed in differentiated muscle satellite cells and its total expression may therefore be diluted in total muscle homogenate. In line with this it has been recently shown in rodents that GLP-1 promotes muscle glucose uptake independently of its incretin action [24] and recruits microvasculature and glucose use in muscle [6]. Previous receptor binding studies have provided evidence that GLP-1R in muscle is functionally different than that found in the pancreas [8]. More specifically, GLP-1R binding studies in rat muscle have shown that muscle GLP-1R is not dependent on cAMP for its activity [7]. However, as the GLP-1R is well expressed in myocytes (Figure 1) it is likely that the receptor plays some role in muscle metabolism. It is important to note that the expression of GLP-1R was suppressed by differentiation of muscle satellite cells in high glucose conditions in the basal state. In rat islets, hyperglycemia has also been shown to decrease gene expression of the GLP-1R by approximately 50% [25]. However, in our model GLP-1R expression remains relatively high and the hyperglycemia-induced decrease is unlikely to explain the loss in GLP-1 mediated glucose uptake.

We have provided strong evidence that exogenous GLP-1 treatment of myocytes promotes glucose uptake and subsequent glycogen storage under normal (5 mM) glucose conditions. These observations have been previously made by one other group (12,10,9) however, the mechanism by which GLP-1 acted and inconsistencies within GLP-1 doses was a limiting factor in helping us understand the potential role of GLP-1 in human skeletal muscle. Importantly our findings support these previous studies that GLP-1-mediated glucose metabolism in muscle is dependent on PI3K activity (Fig 6). In pancreatic beta-cells, GLP-1 has been reported to exert effects on proliferation, DNA synthesis and cell survival in a PI3K-dependent manner [26], [27]. In INS-1 cells, GLP-1 dose dependently increases PI3K activity reaching a maximal response at 10 nM GLP-1 [27] and is non-additive to the glucose effect on PI3K. Furthermore, inhibition of PI3K led to suppressed DNA synthesis by GLP-1 in INS-1 cells [27]. Interestingly, GLP-1 has also been shown to increase the phosphorylation of FOXO1 via a EGFR/PI3K dependent mechanism [28]. It is therefore not surprising that in myocytes GLP-1-mediates its effects on glucose metabolism in a PI3K dependent manner. In disagreement with previous findings [10], [12] we do not find any effect of GLP-1 treatment on PKB/Akt phosphorylation at either Thr^308^ or Ser^473^ (Fig 3 A–C) or that of downstream GSK3 (Fig 3D). This latter finding suggests that the GLP-1-PI3K signaling in skeletal muscle occurs independently of activation of critical components of the insulin signaling cascade. A potential candidate for PI3K-dependent, PKB-independent glucose uptake is PKCζ. PKC zeta has been shown to play a role in glucose uptake independently of PKB [29], [30]. The lab of Villanueva-Penacarillo have shown that inhibition of PKC's (using non-specific pharmacological inhibitors) leads to a suppression of GLP-1-mediated glucose metabolism in muscle (10,12). However, this group did not measure atypical PKC phosphorylation status in cells treated with GLP-1 and therefore the contribution of PKC to this mechanism is unclear. Importantly, in this study we saw no activation of PKC in myocytes incubated with GLP-1 and additionally, inhibition of PI3K had no effect on basal PKC phosphorylation. These findings therefore do not support a role of atypical PKC's in GLP-1-PI3K-mediated glucose metabolism in skeletal muscle.

Our finding that insulin and GLP-1 mediated glucose uptake is impaired by hyperglycemia may indeed be confounded by the trend (Fig 1A) towards increased basal glucose uptake under hyperglycemic conditions. We noted that this trend was significant (P = 0.01) in one experiment (Fig 6 A and B). Elevated basal glucose uptake may be explained by the increase in basal GLUT1 mRNA expression in cells differentiated in hyperglycemic conditions (Fig 4E) and is likely a compensatory mechanism to accommodate for elevated exogenous glucose levels. Additionally, we observed that the elevated basal glucose uptake during hyperglycemia was significantly attenuated by wortmannin treatment suggesting a role for PI3K. This finding is supported by literature indicating that high glucose alone is able to increase PI3K activity in pancreatic beta-cells [26]. In our hands increased glucose uptake (in hyperglycemic conditions) was not accompanied by increased basal PKB Ser^473^ or Thr^308^ phosphorylation (Figure 1) suggesting that hyperglycemia-induced increases in (PI3K-dependent) glucose uptake in the basal state also occurs through a PKB/Akt-independent mechanism. A number of studies have shown that hyperglycemia increases glucose transport through the activation of diacylglycerol (DAG) sensitive PKCs (novel and conventional), particularly through PKCβII [31], [32]. In line with this hyperglycemia promotes *de novo* DAG synthesis through the phosphatidic acid pathway [33] leading to cross talk between PKC and PI3K [34]. Importantly, PI3K has also been shown to promote DAG production through activation of phospholipase D [35]. Therefore in this study it is possible that increased basal glucose uptake under hyperglycemic conditions occurs through PI3K-induced DAG synthesis and subsequent PKC activation.

Increased non-insulin-dependent glucose disposal under hyperglycemic conditions has also been observed *in vivo* at the whole body level [36]. Physiologically, this is important as impaired insulin-mediated glucose disposal in subjects with chronic hyperglycemia would indicate a loss of glycemic control in postprandial periods. Interestingly, insulin-stimulated PKB and downstream GSK-3 phosphorylation were unaffected by high glucose conditions. One interpretation of this is that the defect in insulin-mediated glucose uptake under these conditions occurs at a more distal part of the insulin signaling pathway. It is also possible that increased basal glucose uptake masks the effect of insulin and GLP-1 on glucose uptake due to a maximal uptake capacity being reached rendering the cells insulin resistant in terms of glucose uptake potential but still responsive to insulin at the level of PKB phosphorylation. However, despite the increase in basal glucose uptake, the cells are still insulin-resistant (and GLP-1 resistant) as they are unable to increase glucose uptake capacity in response to hormones. Interestingly, in this study we observed a significant increase in total cell lysate GLUT4 protein in myocytes (cultured under normal glucose conditions) when treated with GLP-1 doses that promoted glucose uptake and glycogen synthesis (Figure 4C and D). This finding suggests that GLP-1 plays a role in the regulation of GLUT4 processing but is not indicative of GLUT4 translocation to the membrane, which due to cell number limitations in such human satellite cell studies could not be measured in this study. Following from this, as insulin is known to promote increased translocation and insertion of GLUT4 into the plasma membrane it is unlikely that insulin treatment would promote increased GLUT4 protein levels. In order to examine this mechanism we also measured GLUT4 expression at the mRNA level (data not shown). Under normal glucose conditions, GLP-1 treatment had no effect on GLUT4 mRNA expression (P = 0.4). In support of GLP-1 regulation of GLUT4 expression it has been reported that treatment of adipocytes with GLP-1 increases glucose uptake and protein expression of both GLUT4 and GLUT2 in total cell lysates [37]. This article hypothesized that increased GLUT4 protein in response to GLP-1 reflects a priming of the cells for subsequent insulin stimulation. The complex regulation of GLUT4 transit occurs within 20–40 minutes with the initiation of GLUT storage vesicles being the longest step (10–20 min) [38]. Additionally, GLUT4 is slowly recycled to the plasma membrane in the basal state therefore it could be possible that GLP-1 in some way increases the amount of available GLUT4 that can be recycled to the plasma membrane resulting in increased glucose uptake. Previous work in 3T3-L1 adipocytes [39] and C2C12 cells [40] has shown that a reduction of the expression (and/or translocation) of GLUT4 leads to reduced glucose uptake, and occurs independently of PKB phosphorylation. In accordance with these previous findings, we have shown that hyperglycemic (22.5 mM) conditions impair GLP-1-mediated total GLUT4 protein level, GLP-1 and insulin-stimulated glucose uptake without inhibiting insulin-stimulated PKB phosphorylation. Potentially, this could be due to a suppression of key adapter proteins, resulting in fewer GLUT4 storage vesicles and reduced cytoplasmic GLUT4. However, hyperglycemia has also been shown to modulate expression profiles of a number of microRNAs (miRNAs) in diabetic [41] and obese [42] rats resulting in suppressed glucose metabolism and GLUT4 expression. To fully understand how GLP-1 increases GLUT4 expression and how hyperglycemia suppresses this effect, further research is required an area that we are continuing to investigate in cellular and human models.

## Conclusions

In conclusion, our present work demonstrates that myocytes express the GLP-1 receptor at the protein level and that differentiation of human satellite cells in high glucose containing media renders the cells both insulin and GLP-1-resistant suppressing both GLP-1 and insulin-stimulated glucose uptake and glycogen synthesis. This work highlights the importance of physiological glucose levels when differentiating satellite cells into myocytes and provides evidence that hyperglycemia may promote both insulin and GLP-1 resistance in skeletal muscle. Additionally, we have shown that GLP-1 promotes glucose uptake in a PKB-independent, PI3K-dependent mechanism in myocytes potentially by increasing GLUT4. The main findings from this study are summarized in Figure 7.
